# Preparation and Evaluation of Long-Acting Injectable Levocetirizine Prodrug Formulation

**DOI:** 10.3390/pharmaceutics17070806

**Published:** 2025-06-21

**Authors:** Jun-hyun Ahn

**Affiliations:** Department of Biopharmaceutical Engineering, College of Life Science and Nano Technology, Hannam University, Deajeon 34054, Republic of Korea; ajh@hnu.kr

**Keywords:** levocetirizine, prodrug, long-acting injectable, depot formulation, allergic rhinitis, ester hydrolysis, pharmacokinetics

## Abstract

**Background/Objectives**: Levocetirizine (LCZ) is a second-generation antihistamine with minimal central nervous system effects. However, its short half-life necessitates daily dosing, potentially reducing adherence in pediatric populations. This study aimed to develop a long-acting injectable LCZ formulation by synthesizing lipophilic prodrugs and evaluating their physicochemical stability, enzymatic hydrolysis, and pharmacokinetics in vivo. **Methods**: Two prodrugs of LCZ, LCZ decanoate (LCZ-D) and LCZ laurate (LCZ-L), were synthesized via esterification with alkyl alcohols. The compounds were characterized using NMR, FT-IR, and DSC. Prodrugs were formulated with an oil-based vehicle (castor oil and benzyl benozate), and their hydrolysis was evaluated using porcine liver esterase (PLE) and rat plasma. Pharmacokinetic profiles were assessed in Sprague Dawley rats after oral or intramuscular administration. Stability was tested at 25 °C, 40 °C, and 60 °C for 6 weeks. **Results**: LCZ-D and LCZ-L exhibited first-order hydrolysis kinetics, with rates following the order of PLE (2.0 > 0.5 units/mL) > plasma > PLE (0.2 units/mL). The C_max_ of LCZ-D and LCZ-L were 13.95 and 5.12 ng/mL, respectively, with corresponding AUC_0–45d_ values of 6423.12 and 2109.22 h·ng/mL. Formulations containing excipients with lower log *P* values led to increased systemic exposure. All formulations maintained therapeutic plasma concentrations for over 30 days. The inclusion of the antioxidant BHT (0.03% *v*/*v*) improved oxidative stability, reducing degradation at 60 °C from 4.72% to 1.17%. **Conclusions**: All formulations demonstrated potential for the long-acting delivery of LCZ, maintaining therapeutic plasma levels for over 30 days. Moreover, the release behavior and systemic exposure could be effectively modulated by excipient selection.

## 1. Introduction

Allergic rhinitis (AR) is a common non-infectious inflammatory disease around the world. In the ISAAC phase III study (consisting of data from 236 centers in 98 countries), AR prevalence increased from 8.5% in individuals aged 6–7 years to 14.6% in those aged 13–14 years. AR in people aged 20–44 ranged from 11.8% in Oviedo (Spain) to 46.0% in Melbourne (Australia) [1]. On average, 83.14 persons per a population of 1000 are diagnosed with AR in South Korea [2,3]. The prevalence of AR among South Korean children under the age of 10 has been reported to be notably high, reaching 38.4%, and continues to show an increasing trend [4]. This trend in prevalence continues to impose a growing socioeconomic burden [5,6].

According to the Allergic Rhinitis and its Impact on Asthma (ARIA) guidelines, AR can be classified based on its duration (intermittent or persistent) and severity (mild to moderate or severe) [7,8]. Intermittent allergic rhinitis is defined as symptoms occurring less than 4 consecutive days/week or less than 4 consecutive weeks/year. Persistent allergic rhinitis is defined as symptoms occurring more often than 4 consecutive days/week and for more than 4 consecutive weeks/year [9]. AR is triggered by hypersensitivity to allergens. Its symptoms are primarily mediated by the release of histamine from mast cells, which promotes immune cell recruitment, while goblet cell hyperplasia contributes to excessive watery secretions [10].

For symptomatic relief, oral antihistamines and intranasal corticosteroid sprays are recommended. Intranasal delivery offers superior efficacy in alleviating nasal congestion and sneezing; however, limitations such as poor portability and a bitter aftertaste may reduce pediatric patient compliance [11]. In addition, antihistamines are favored over intranasal formulations owing to their comparatively lower cost [12].

First-generation antihistamines are well known to cross the blood–brain barrier (BBB) and act on H1 receptors involved in the regulation of the sleep–wake cycle, thereby causing drowsiness as a common adverse effect. Consequently, they are not recommended for long-term use. In contrast, second-generation or later antihistamines are preferred due to their high selectivity for peripheral H1 receptors and reduced anticholinergic activity [13]. Levocetirizine (LCZ), categorized as a second-and-a-half generation antihistamine, represents a refined active pharmaceutical ingredient from which dextrocetirizine (S-enantiomer), implicated in adverse effect profiles, has been removed. In the therapeutic dose range for the treatment of allergic rhinitis and urticaria, LCZ exhibits a brain H1 receptor occupancy of approximately 8.1%, a threshold generally regarded as clinically insignificant when below 10%. Moreover, clinical studies comparing levocetirizine and fexofenadine have demonstrated no significant differences in sedation-related adverse effects [14].

The sustained release of drugs from specific sites offers clinical advantages in the treatment and management of various diseases, including central nervous system (CNS) disorders. Researchers are striving to design and develop more innovative and efficient therapeutic approaches for a wide range of diseases [15]. Although allergic rhinitis does not directly affect survival and may appear less harmful compared to other medical conditions, pediatric and adolescent patients with persistent allergic rhinitis often face challenges in maintaining consistent medication adherence [16,17]. Poor medication adherence may lead to sleep disturbances and impaired academic performance [18]. Although intravenous QUZYTTIR^®^ (Cetirizine HCl) has been approved by the FDA in 2019 for the treatment of acute urticaria as an alternative to oral administration in pediatric patients, it remains insufficient for patients requiring long-term therapy. Additionally, intramuscular injections have been widely accepted for neonatal vaccination and are considered feasible for administration in the deltoid or thigh muscles of pediatric patients aged 3 to 18 years [19].

The use of poly(lactic-co-glycolic acid) represents a widely established technology for transforming oral pharmaceuticals into long-acting injectable formulations. By 2022, a total of 25 PLGA-based products had received FDA approval [20,21]. However, for water-soluble drugs such as LCZ 2HCl, the encapsulation efficiency of PLGA microparticles is expected to be markedly low, resulting in inherent limitations in the drug loading capacity [22]. Technologies utilizing self-assembled lipid nanoparticles (LNPs) enable the encapsulation of water-soluble drugs with a high encapsulation efficiency, such as mRNA, and facilitate targeted delivery to specific biological sites. However, patent restrictions regarding the specific lipid used and the high production costs remain barriers to pharmaceutical development [23].

From the past to the present, one widely utilized strategy for formulating long-acting injectables involves the prodrug approach when peptides or drugs possess functional groups such as hydroxyl (-OH), carboxyl (-COOH), or amine. In marketed formulations, saturated fatty chains are commonly employed as promoieties, and the introduction of ester bonds is selected to enable appropriate enzymatic cleavage. This strategy is considered safe because acetylcholinesterase and butyrylcholinesterase are abundantly present in human muscle tissue and body fluids, and the cleavaged fatty chains can be utilized in energy metabolism [24,25]. This prodrug approach offers a cost-effective method for modulating physicochemical properties, permeability, and the duration of drug action. The duration of action can be regulated by altering a chain length, which in turn indirectly affects the pharmacokinetic parameters of the parent drug, including its absorption, distribution, metabolism, and excretion [26].

This research aims to synthesize prodrugs of LCZ with fatty alkyl alcohols of varying chain lengths for intramuscular injection, to achieve the sustained release of the parent drug over a month. Additionally, we evaluated the in vitro physicochemical properties of the prodrugs and aimed to formulate an oil-based depot system that enables intramuscular injection. The selected formulations were administered to SD rats and the pharmacokinetic parameters and thermal stability were evaluated.

## 2. Materials and Methods

### 2.1. Materials

LCZ 2HCl was kindly provided by Daewon Pharmaceutical (Seoul, Republic of Korea). Benzyl benzoate (BB), benzyl alcohol (BA), 1-decanol, 1-dodecanol, polysorbate 80 (TW80), dimethylsulfoxide (DMSO), porcine liver esterase (PLE) and Butylated hydroxytoluene (BHT) were purchased from Sigma Aldrich (St. Louis, MO, USA), kept in appropriate storage conditions. Super Refined^TM^ castor oil (CO) was supplied by Croda (East Yorkshire, UK). Sodium bicarbonate, sodium sulfate, sulfuric acid, methanol, ethylacetate, acetonitrile (ACN), chloromethane (MC), formic acid, ammonium formate and the other chemical reagents were obtained from Samchun chemicals (Seoul, Republic of Korea).

The synthetic reaction was monitored by precoated Silicagel 60 thin-layer chromatography, and the Silicagel 60 (0.040–0.063 mm) used for open column chromatography was purchased from Merck KGaA (Darmstadt, Germany). Deuterated chloroform (CDCl_3_) and Tetramethylsilane (TMS) were used for ^1^H-NMR. Sprague Dawley (SD) rats and rat plasma were obtained from Orientgenia (Osan, Korea), and levocetirizine-d_4_ was used as an internal standard for the LC–MS/MS analysis. All chemicals used were analytical grade or higher, and in-house purified water from Milli-Q system (Millipore, Molsheim, France) was used throughout the study.

### 2.2. Synthesis of Levocetirizine Prodrugs

**Figure 1:**
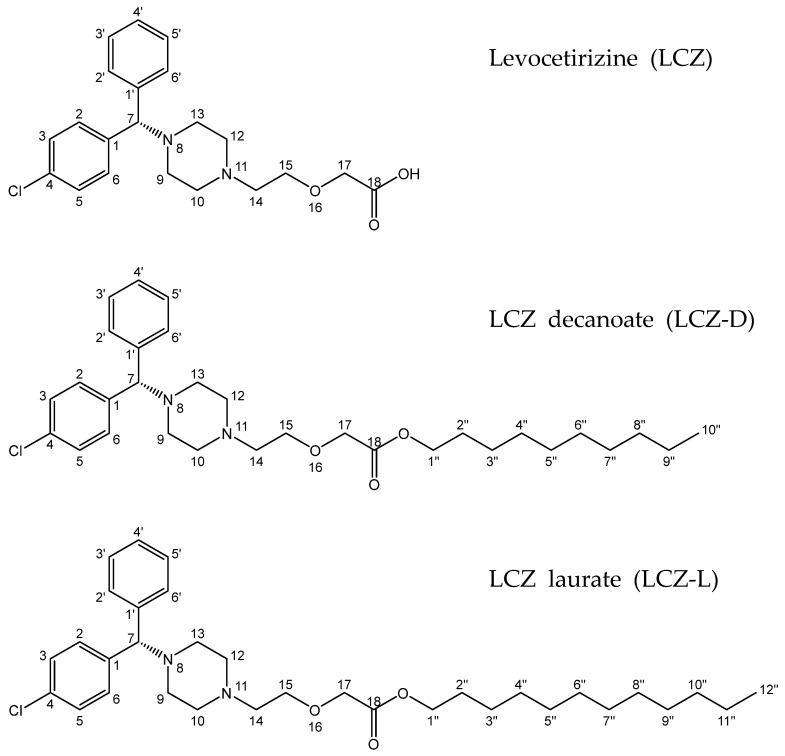
Chemical structures of levocetirizine and its prodrug derivatives. The ester linkage introduced during conjugation is readily hydrolyzed under physiological conditions.

In the round flask equipped with a reflux condenser, the powder of LCZ 2HCl (5.08 g, 11 mmol) was mixed with alkyl alcohol (3 Eq.), sulfuric acid (0.2 mL) and tetrahydrofuran. The reaction mixture was stirred at 90 °C in a hot plate magnetic stirrer overnight. After the completion of Fischer esterification [27], the flask was allowed to cool to room temperature and ethylacetate was added (50 mL). The product was transferred into a separating funnel and then 7 mL of saturated sodium bicarbonate solution was added to the funnel to neutralize the inorganic acid. The organic phase was washed out with the brine solution and sodium sulfate. The remaining organic phase was concentrated using a rotary solvent evaporator. The crude compound was loaded to Silicagel 60, packed in a glass column. To remove excess alkyl alcohol in the compound, the column was washed out by 300 mL of MC two times. The final product was obtained by the solvent (MC:MeOH = 1:9). The columned product was identified by TLC and rotary-evaporated for 12 h to remove the residual solvent. The molecular weights of the LCZ prodrugs (Figure 1) were 529.16 g/mol and 557.22 g/mol. The clog *P* was calculated using ChemDraw 15.1 software.

### 2.3. Nuclear Magnetic Resonance Spectroscopy

The ^1^H NMR spectra of the final compounds were recorded on a JNM-ECZ600R NMR spectrometer operating at 600 MHz (JEOL, Tokyo, Japan). The 1H NMR spectra were recorded using CDCl_3_ as a solvent, and chemical shifts (δ) were reported in ppm relative to TMS as an internal standard. Multiplicities are indicated as s (singlet), d (doublet), t (triplet), q (quartet), m (multiplets), dd (doublet of doublets), and td (triplet of doublets), and coupling constants (J) are reported in hertz (Hz). The NMR data was analyzed using MestReNova 14.3.3 software.

### 2.4. Fourier Transformed Infrared Spectroscopy

The spectra of LCZ 2HCl, LCZ-D LCZ-L and the physical mixture (PM) were prepared using the Cary 630 device equipped with an ATR accessory (Agilent, Santa Clara, CA, USA). The data were collected by placing a small amount of powder or liquid on the diamond crystal and pressing this with the attached arm to remove air in the sample. The data collection parameters were a scanning range of 650–4000 cm^−1^ with a resolution of 4 cm^−1^ and 32 scans. The background was measured on every sample. Microlab (Agilent, Santa Clara, CA, USA) and Excel (Microsoft, Redmond, Washington, DC, USA) were used to collect and analyze the data.

### 2.5. Differential Scanning Calorimetry

The instrument used for differential scanning calorimetry (DSC) analysis was DSC 214 Polyma (Netzsch, Selb, Germany). Aqueous samples (10 mg) were prepared in sealed aluminum capsules. The samples were scanned at 25–250 °C at a rate of 10 °C/min to cover the melting point of LCZ 2HCl. Nitrogen gas was purged at 20 psi pressure at a rate of 30 mL/min to prevent oxidation reactions.

### 2.6. Hydrolysis of Prodrug in Porcine Liver Esterase (PLE)

The ester bond cleavage assay was adapted from reference [28], with minor modifications, and carried out using PLE to evaluate the hydrolytic ratio (*n* = 3). Briefly, the LCZ prodrug (1 mg, LCZ 2HCl Eq.) was dissolved in 10% DMSO solution (4 mL) containing PLE (2.0, 0.5 and 0.2 unit/mL) and incubated at 37 °C in a shaking chamber (100 rpm). At 0, 1, 2, 4, 6 and 24 h, the aliquot (100 μL) was collected using a 28 G syringe, and then immediately diluted 10-fold with a solvent composed of Acetonitrile:DW = 90:10 (*v*/*v*) to stop enzymatic action. The elution was replenished with an equal concentration and volume of fresh esterase solution. The diluted samples were filtered through a double 0.45 μm PTFE syringe filter. The filtrates were analyzed for their prodrug and LCZ content by HPLC to determine the conversion of the prodrug to LCZ.

### 2.7. Hydrolysis of Prodrugs in Rat Plasma

Stock solutions of LCZ and its prodrugs (10 mg/mL, LCZ Eq.) were prepared in DMSO. First, 100 μL of stock solution was added to 3.9 mL of heparinized rat plasma (*n* = 3). The samples were incubated at 37 °C in a shaking chamber (100 rpm). At 0, 1, 2, 4, 6 and 24 h, the aliquot (100 μL) was collected, and then immediately diluted 10-fold with a solvent composed of Acetonitrile:DW = 90:10 (*v*/*v*) to precipitate the protein. An equal volume of fresh rat plasma was replenished after each sampling. The samples were centrifuged at 5000 rpm for 10 min and the supernatants were filtered through a double 0.45 μm PTFE syringe filter. The filtrates were analyzed for their prodrug and LCZ content by HPLC to determine the conversion of the prodrug to LCZ.

### 2.8. Preparation of Formulation

All formulations were prepared using a simple mixing method (Table 1). Briefly, the LCZ 2HCl solution (0.15 mg/mL) for oral administration was prepared immediately before dosing. LCZ prodrugs (150 mg/g, LCZ 2HCl Eq.) were mixed with an organic phase consisting of benzyl benzoate and castor oil (6:4, *v*/*v*) using a magnetic stirrer for 3 h [29,30]. All formulations were filtered through a 0.2 μm PTFE syringe filter for sterilization and confirmed by measuring the drug content prior to the in vivo study.

### 2.9. Pharmacokinetic Study

The pharmacokinetic profiles of LCZ and its prodrugs were evaluated in male SD rats (*n* = 4). All animals were acclimatized in a controlled environment for a minimum of three days prior to drug administration. Throughout the experimental period, animals were maintained under ad libitum feeding conditions. Drug administration was limited to rats with a body weight of 200 ± 10 g. F1 was administered orally at a dose of 0.5 mg/kg once daily for seven consecutive days. Blood samples were collected from the rats on day 1 and day 7 at 0, 0.5, 1, 2, 4, and 6 h. On the intermediate days (days 2–6), a single blood sample was collected prior to administration. The F2–6 was administered as a single intramuscular injection at a dose of 15 mg/kg into the left thigh muscle. Blood sample was collected at 0, 2, 4, and 6 h, and at 1, 3, 5, 7, 10, 14, 21, 30, and 45 days.

All blood samples were collected with commercial K_2_EDTA-coated tubes and centrifuged at 5000× *g* for 10 min within 30 min of collection. The plasma was immediately transferred to 2 mL Eppendorf tubes and stored on ice during processing. Samples were then stored at −70 °C in a freezer until analysis by LC–MS/MS.

### 2.10. Stability Test

The prepared formulations F3 and F7 were sterilized using a 0.2 μm PTFE syringe filter and filled into pre-washed, 10 mL lyophilized vials containing 200 mg of formulation. The vials were sealed with a butyl rubber stopper under 70 kPa pressure using a freeze dryer, and aluminum capping was performed to ensure proper sealing. The samples were placed in three separate dry ovens set to 25 °C, 40 °C, and 60 °C to maintain the storage conditions. The contents of the prodrug and LCZ were evaluated at five predetermined time points—0, 1, 2, 4, and 6 weeks—using the HPLC method.

### 2.11. HPLC, LC-MS/MS Condition and Data Analysis

Esterase, plasma hydrolysis and chemical stability studies were performed using the HPLC method. The HPLC equipment used was Infinity 1260 (Agilent, CA) fitted with a quaternary pump, an autosampler, a UV detector set at a wavelength of 230 nm and a column compartment maintained at 20 °C. The sample was eluted from a Fortis C18 (150 × 4.6 mm, 5 μm particle size) (Fortis, Neston, UK) analytical column using Acetonitrile:distilled water:sulfuric acid (90:9.6:0.4, *v*/*v*). The mobile phase was pumped isocratically at a flow rate of 1 mL/min with a 20 μL injection volume.

The quantification of LCZ and LCZ prodrugs in the PK samples was analyzed on a Acquity UPLC (Waters, Milford, MA, USA) instrument coupled with a 4000 QTRAP triple quadrupole mass spectrometer operating in positive multiple-reaction monitoring (MRM) mode. The plasma samples stored at −70 °C were thawed at room temperature prior to analysis. A 50 μL aliquot of plasma was mixed with 50 μL of methanol containing levocetirizine-d_4_ (10 ng/mL) as an internal standard. The mixture was vortexed briefly and diluted to an appropriate concentration for analysis. The separation of the processed plasma samples was performed on a CAPCELL PAK C18 MGII (3.0 × 75 mm, 3.0 μm, Osaka Soda, Osaka, Japan) instrument maintained at 20 °C in the column oven. The mobile phase consisted of 10 mM of ammonium formate (0.1% formic acid):Acetonitrile (0.1% formic acid) = 35:65 (*v*/*v*), flowing at 0.25 mL/min with an 8 μL injection volume. The detection parameters were LCZ (*m*/*z* precursor ion 389.2 → product ion 201.0) and LCZ-d_4_ (*m*/*z* 393.3 → 201.0). The calibration range was from 0.2 to 100 ng/mL. The precision and accuracy of the samples met the requirement of ±15% for the nominal concentration. The pharmacokinetic parameters were calculated using the Phoenix WinNonlin 6.4 and Microsoft Excel 2024 software.

## 3. Results

### 3.1. Nuclear Magnetic Resonance Spectroscopy

The ^1^H-NMR (600 MHz) of LCZ-D (Figure S1) (Table S1) showed δ and J values for protons at 7.39–7.32 (m, 4H, chlorophenyl), 7.29–7.20 (m, 4H, aromatic proton), 7.20–7.14 (m, 1H, aromatic proton), 4.20 (s, 1H, C7 proton), 4.12 (t, *J* = 6.8 Hz, 2H, C1″ proton), 4.07 (s, 2H, C17 proton), 3.65 (t, *J* = 5.6 Hz, 2H, C15 proton), 2.62 (t, *J* = 5.6 Hz, 2H, C14 proton), 2.48 (br d, *J* = approx. 70.0 Hz, 8H, piperazine proton), 1.65–1.58 (m, 2H, C2″ proton), 1.37–1.20 (m, 14H, C3″–C9″ proton), and 0.88 (t, *J* = 7.0 Hz, 3H, C10″ proton).

The ^1^H-NMR of LCZ-L (Figure S1) (Table S2) showed δ and J for various protons at δ 7.38–7.32 (m, 4H, chlorophenyl proton), 7.29–7.20 (m, 4H, phenyl proton), 7.20–7.14 (m, 1H, phenyl proton), 4.20 (s, 1H, C7 proton), 4.12 (t, *J* = 6.8 Hz, 2H, C1″ proton), 4.08 (s, 2H, C17 proton), 3.66 (t, *J* = 5.7 Hz, 2H, C15 proton), 2.63 (t, *J* = 5.7 Hz, 2H, C14 proton), 2.48 (br d, 8H, piperazine proton), 1.65–1.58 (m, 2H, C2″ proton), 1.27–1.24 (m, 18H, C3″–C11″ proton), and 0.88 (t, *J* = 7.0 Hz, 3H, C12″ proton).

### 3.2. Fourier Transform Infrared Spectroscopy

**Figure 2:**
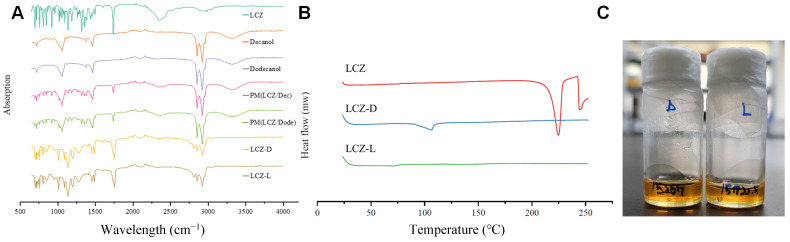
Physicochemical properties of levocetirizine, LCZ-D and LCZ-L. (**A**) FT-IR spectra of levocetirizine, decanol, dodecanol, physical mixture and prodrugs. (**B**) Differential scanning calorimetry image. (**C**) Appearance of the prodrug at room temperature.

The FT-IR spectra of the various samples are presented in Figure 2A. LCZ exhibited characteristic carbonyl (C=O) stretching at 1760 cm^−1^, indicative of a carboxylic acid functional group [31]. For decanol and dodecanol, alkyl (C–H) stretching were observed at 2920 cm^−1^, along with (O–H) stretching of the alcohol group at 2850 cm^−1^. In the physical mixture of LCZ with the alcohols, all characteristic peaks were retained. However, in the synthesized LCZ prodrugs, shifted ester (C=O) stretching was observed at 1750 cm^−1^, along with a C–O stretching band at 1125 cm^−1^. The alcohol (O–H) stretching at 2850 cm^−1^ disappeared, indicating ester bond formation between LCZ and the alcohol moieties.

### 3.3. Differential Scanning Calorimetry (DSC) and Appearance

The DSC thermogram overlays of LCZ and its synthesized prodrugs are presented in Figure 2B. LCZ exhibited an endothermic peak between 215 and 225 °C, corresponding to its melting point, consistent with previous reports [32]. The LCZ prodrugs did not display any melting point and existed as viscous, transparent yellow liquids with an oil-like consistency (Figure 2C). These prodrugs did not solidify even under storage conditions at −20 °C. Therefore, they were stored at −70 °C for subsequent experiments.

### 3.4. LCZ Prodrug Hydrolysis

The water solubility of the prodrug, as determined by a preliminary test, was found to be below the detection limit of the HPLC method (0.2 μg/mL). Therefore, DMSO was selected as a co-solvent to facilitate mixing with water without inhibiting the enzymatic conversion of the prodrug. It has been reported that the use of 10% DMSO maintains more than 90% of PLE enzymatic activity [33]. Under these solvent conditions, the solubility of both LCZ and its prodrugs sufficiently covered the experimental concentration range, and no degradation was observed for up to 24 h, confirming their stability. The selected co-solvent system provided an optimal balance between solubility enhancement and enzymatic compatibility.

**Figure 3:**
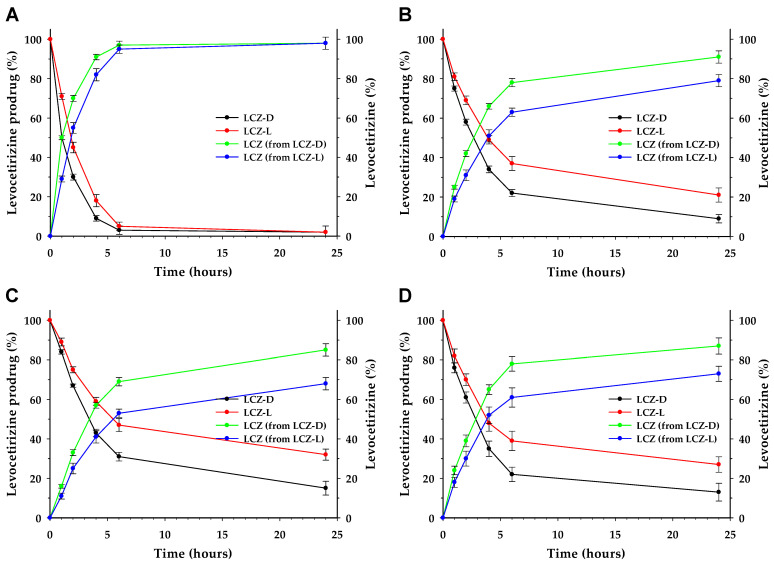
Enzymatic conversion of LCZ prodrugs to free LCZ in 10% DMSO solution from (**A**) PLE at 2.0 units/mL, (**B**) PLE at 0.5 units/mL, (**C**) PLE at 0.2 units/mL, and (**D**) rat plasma. Data of mass balance are presented as mean ± S.D (*n* = 3).

As shown in Figure 3A–D, the enzymatic hydrolysis of LCZ-D and LCZ-L was assessed using PLE at concentrations of 2.0, 0.5, and 0.2 unit/mL, as well as in rat plasma. The half-life (T_1/2_) was defined as the time required for the 50% degradation of each prodrug. At 2.0 unit/mL PLE, LCZ-D showed rapid hydrolysis with T_1/2_ estimated at 1 h, whereas LCZ-L showed slower degradation with T_1/2_ estimated at 2 h. At 0.5 unit/mL, the T_1/2_ values were extended to 3 h for LCZ-D and 4 h for LCZ-L. A further reduction to 0.2 unit/mL resulted in T_1/2_ values of 4 and 5 h, respectively. In rat plasma, the hydrolysis was slightly slower than that observed at 0.5 unit/mL PLE, but the relative degradation profiles of the two prodrugs were maintained. In all conditions, levocetirizine was quantitatively released, with near-complete mass balance, indicating the efficient conversion of both prodrugs to the parent compound.

**Figure 4:**
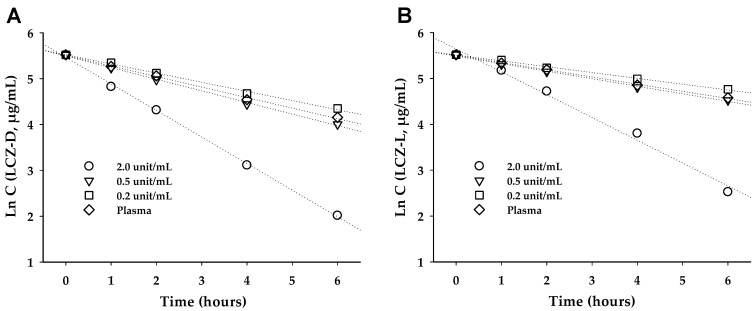
First-order kinetics of LCZ prodrugs in PLE (2.0, 0.5, 0.2 unit/mL) and rat plasma during 6 h: (**A**) LCZ-D, (**B**) LCZ-L.

In cases where the substrate concentration is low or due to other undefined factors, enzyme kinetics may deviate from the Michaelis–Menten model and instead follow first-order kinetics [34]. The first-order kinetics are shown in Figure 4. The first-order rate constants (kobs) of LCZ-D at 2.0, 0.5, and 0.2 units/mL for PLE and rat plasma were 0.580, 0.253, 0.201, and 0.229 h^−1^, respectively (R^2^ = 0.980), while those of LCZ-L were 0.498, 0.165, 0.127, and 0.157 h^−1^, respectively (R^2^ = 0.970). The hydrolysis rate for LCZ prodrugs followed the following order: PLE 2.0 > 0.5 > plasma > 0.2 units/mL. The linearity of the regression fits supports the validity of the first-order kinetic model and supports the presumption that the drug concentration (0.54 mM LCZ Eq.) used in the experiment was below the k_m_ value [35,36,37].

### 3.5. Pharmacokinetics

**Figure 5:**
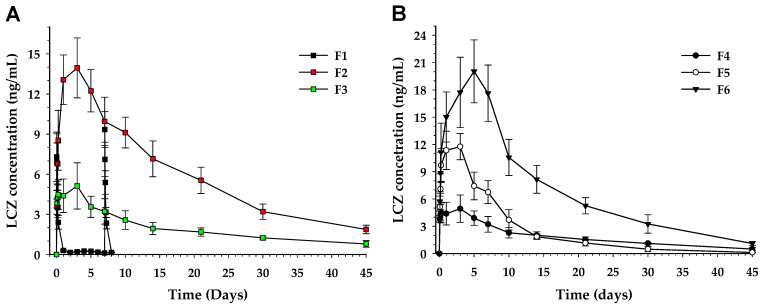
Plasma concentration–time profiles of LCZ in rats following the administration of formulations (**A**) F1 (oral) and F2–F3 (intramuscular); (**B**) F4–F6 (intramuscular). Data are presented as mean ± S.D. (*n* = 4).

Pharmacokinetic evaluations were conducted in male Sprague Dawley rats to compare oral and intramuscular administration (Figure 5). Following the daily oral administration of F1 (LCZ solution), the C_max_ values on Day 1 and Day 7 were 7.31 ± 1.85 and 9.35 ± 1.32 ng/mL, respectively (Table 2). The corresponding AUC_0–1d_ and AUC_0–7d_ values were 52.72 ± 11.86 and 47.78 ± 8.10 h·ng/mL. T_max_ was consistently observed at 30 min for both time points, and no evidence of drug accumulation was observed during the 7-day repeated administration. In contrast, a single intramuscular injection of F2 (LCZ-D) into the thigh muscle resulted in a C_max_ of 13.95 ± 2.24 ng/mL, with T_max_ occurring on Day 3. The AUC_0–45d_ was 6423.12 ± 1063.11 h·ng/mL, representing a 4.06-fold increase compared to the theoretical cumulative AUC_0–30d_ of F1 administration. F3 (LCZ-L) exhibited a C_max_ of 5.12 ± 1.73 ng/mL, with a T_max_ also on Day 3, and an AUC_0–45d_ of 2109.22 ± 497.96 h·ng/mL, which was 1.33-fold higher than the theoretical AUC_0–30d_ of F1. Moreover, the mean residence times (MRTs) of F2 and F3 were extended by 2.38- and 2.47-fold, respectively, compared to that of F1 normalized to a 30-day period. These results indicate that both LCZ-D and LCZ-L prodrugs provide sustained systemic exposure and that LCZ-D appears to offer more favorable outcomes than LCZ-L in terms of achieving and maintaining the intended systemic exposure. For F4 (LCZ-L), the formulation was designed with double the drug concentration compared to F3. Although the C_max_ of F4 slightly decreased to 4.94 ± 1.51 ng/mL, there was no notable difference in T_max_ or AUC_0–45d_, suggesting that the physicochemical properties of the prodrug, rather than the drug concentration, may play a dominant role in influencing systemic absorption. For F5 (LCZ-L with 1% TW80), the C_max_ was 11.76 ± 1.44 ng/mL, indicating a 2.30-fold enhancement compared to F3, while T_max_ remained unchanged. However, the plasma concentrations of F5 rapidly declined after reaching C_max_, becoming comparable to F3 by day 14. The AUC_0–45d_ was 2761.46 ± 596.15 h·ng/mL, which is approximately 1.3-fold higher than that of F3. For F6 (LCZ-L with 3% benzyl alcohol), the C_max_ was 20.03 ± 3.45 ng/mL, representing a 2.30-fold increase compared to F3. The T_max_ was slightly delayed to Day 5. The AUC_0–45d_ was 7618.75 ± 1539.69 h·ng/mL, which corresponds to a 3.91-fold increase relative to F3.

The calculated log *P* values of LCZ-D, LCZ-L, Tween 80, benzyl benzoate, and benzyl alcohol were 9.32, 10.38, 4.39, 3.98, and 1.10, respectively. A relatively small difference in the log *P* between LCZ-D and LCZ-L resulted in a substantial difference in systemic exposure. The incorporation of excipients with lower log *P* values further reduced the overall lipophilicity of the formulation, which in turn enhanced systemic exposure.

### 3.6. Stability

During the stability testing of the LCZ prodrugs (Table 3), LCZ was the only degradation impurity detected by the HPLC method, with no other impurities observed. According to the ICH Q3A(R2) and Q3B(R2) guidelines, “impurities that are also significant metabolites present in animal and/or human studies are generally considered qualified” [38]. Since LCZ is the active metabolite of the prodrug in this study, this requirement is considered to apply with reduced stringency. As shown in Figure S2, preliminary forced degradation studies demonstrated that the prodrug formulation is vulnerable to oxidative stress. Therefore, BHT was selected as an antioxidant compatible with lipid-based formulations. After 6 weeks of storage at 60 °C, the F7 (containing 0.03% BHT) showed 1.17% degradation of the prodrug (*n* = 3). This result reflects a 4.03-fold decrease in degradation relative to the F3 formulation (without BHT), which showed 4.72% degradation under identical conditions. Therefore, these results demonstrate that the addition of BHT is effective in suppressing oxidative degradation and contributes to the improved chemical stability of the LCZ prodrug.

## 4. Discussion

This study investigated a long-acting injectable formulation to achieve the sustained release of LCZ, for which prodrugs were synthesized by introducing alkyl chains into the LCZ molecule. These synthesized prodrugs were successfully purified using open column chromatography, and their structures were confirmed by NMR and FT-IR spectroscopy. In vitro drug release profiling using PLE enabled the elucidation of the degradation mechanism of the prodrugs. The two promoieties and two types of excipients were all associated with changes in the log *P* values, and it was observed that even a modest reduction in log *P* significantly increased systemic exposure.

In intramuscular oil-based depot formulations, accurate estimation of the elimination half-life of the prodrug is inherently difficult. This is because the injected oil formulation forms a reservoir in the muscle or adipose tissue [39], where it undergoes slow enzymatic conversion and sustained release over time. In this study, similarly, if the injected formulations had been immediately transferred to the systemic circulation, they would have been degraded within a very short period (6–24 h), unlike orally administered LCZ, which is rapidly cleared from the body. As a result, drug absorption and elimination occur concurrently over an extended period. Therefore, the pharmacokinetic profile observed after T_max_ is best explained by the flip-flop model [40]. LCZ-L exhibited a slower hydrolysis rate in the in vitro enzymatic assay and showed a corresponding reduction in systemic exposure in vivo. These findings suggest a potential correlation between in vitro hydrolysis kinetics and in vivo pharmacokinetic behavior, implying that enzymatic degradation profiles may provide predictive insights into the systemic exposure of long-acting prodrug formulations.

In a previously published study [41], the oral administration of LCZ in children aged 6–11 years yielded a C_max_ of 450 ± 0.1 ng/mL. The EC_50_ values were reported as 1.4 ± 0.1 ng/mL for flare suppression and 16.1 ± 2.2 ng/mL for wheal suppression. In the present study, rats were administered a human equivalent dose (HED) scaled by a factor of six; however, the systemic exposure was lower than that reported in humans. This discrepancy may be attributed to interspecies differences in clearance rates or plasma protein binding [42]. The HED is generally determined by considering the weight, body surface area, and k_m_ values of animals and humans. This calculation is important for determining the no observed adverse effect levels (NOAELs) when a drug is first administered to humans, as well as for predicting human dosing intervals to maintain therapeutic efficacy and minimize toxicity. According to FDA guidance, an allometric scaling factor of 6.2 is recommended for converting rat data to human equivalent doses. However, depending on the drug’s toxicity, a lower starting dose may need to be considered [43,44].

Among the tested formulations, only LCZ-L did not exceed the C_max_ of F1 and exhibited a longer plateau plasma concentration, indicating a potentially safer systemic exposure. Therefore, LCZ-L is considered more favorable for long-acting parenteral delivery, as it maintains therapeutic levels for at least one month without inducing an burst effect. Despite these translational limitations, these findings suggest that LCZ prodrugs could maintain plasma concentrations exceeding the EC_50_ for wheal suppression in humans for at least 30 days.

## 5. Conclusions

In conclusion, this study aimed to develop and evaluate long-acting injectable formulations of LCZ by synthesizing prodrugs with various promoieties and formulating them with lipid-based excipients. Among the tested formulations, LCZ-L showed favorable pharmacokinetic characteristics, maintaining therapeutic plasma concentrations for over 30 days without inducing a burst release. Systemic exposure was effectively modulated through variations in chemical structure and excipient composition by altering the log *P* values. These results support the potential use of LCZ prodrugs for sustained and safe drug delivery.

## Figures and Tables

**Table 1:** Composition of formulations for pharmacokinetics and stability study.

Formulation	Composition			
	Drug		Excipient	
F1	LCZ 2HCl	150.0 mg	Distilled water	850.0 mg
F2	LCZ-D	171.9 mg	BB:CO (6:4)	828.1 mg
F3	LCZ-L	181.0 mg	BB:CO (6:4)	819.0 mg
F4	LCZ-L	181.0 mg	BB:CO (6:4)	319.0 mg
F5	LCZ-L	181.0 mg	BB:CO (6:4), TW80	804.0 mg, 15.0 mg
F6	LCZ-L	181.0 mg	BB:CO (6:4), BA	789.0 mg, 30.0 mg
F7	LCZ-L	181.0 mg	BB:CO (6:4), BHT	818.7 mg, 0.3 mg

**Table 2:** Pharmacokinetic parameters of F1–F6 in rats. Data are presented as mean ± standard deviation (*n* = 4). C_max_: maximum plasma concentration; T_max_: time to reach C_max_; AUC: area under the plasma concentration–time curve.

Formulation	Parameter				
	C_max_ (ng/mL)	T_max_ (day)	AUC_1d_ (h·ng/mL)	AUC_0–30d_ (h·ng/mL)	AUC_0–45d_ (h·ng/mL)
F1	7.31 ± 1.85 (1d) 9.35 ± 1.32 (7d)	0.083	52.72 ± 11.86 (0–1d) 47.78 ± 8.10 (7–8d)	(1581.75 *) (1433.33 *)	-
F2	13.95 ± 2.24	3	-	5510.53 ± 1063.11	6423.12 ± 1063.11
F3	5.12 ± 1.73	3	-	1743.82 ± 497.95	2109.22 ± 497.96
F4	4.94 ± 1.51	3	-	1697.05 ± 434.96	1987.75 ± 434.96
F5	11.76 ± 2.44	3	-	2652.42 ± 596.15	2761.46 ± 596.15
F6	20.03 ± 3.46	5	-	6832.15 ± 1539.69	7618.75 ± 1539.69

* For F1, AUC_0–30d_ was theoretically calculated by multiplying AUC_1d_ by 30.

**Table 3:** Stability profiles of formulations F3 and F7 based on the formation of LCZ as a degradation product under accelerated conditions (25 °C, 40 °C, and 60 °C) during 6 weeks.

Formulation	Condition (°C)	Impurity (=LCZ)				
		0 Week	1 Week	2 Week	4 Week	6 Week
F3	25	0%	0.19%	0.24%	0.35%	0.58%
	40	0%	0.42%	0.60%	0.88%	1.23%
	60	0%	0.65%	1.15%	2.67%	4.72%
F7	25	0%	0.06%	0.07%	0.09%	0.12%
	40	0%	0.09%	0.14%	0.22%	0.31%
	60	0%	0.23%	0.41%	0.73%	1.17%

## Data Availability

The original contributions presented in this study are included in the article/Supplementary Materials. Further inquiries can be directed to the corresponding author.

## Supplementary Materials

The following supporting information can be downloaded at: https://www.mdpi.com/article/10.3390/pharmaceutics17070806/s1, Figure S1. ^1^H NMR spectra of LCZ-D and LCZ-L acquired in CDCl_3_ at 600 MHz. Signal assignments for key protons are labeled. Figure S2. HPLC chromatogram of LCZ-L after 3 h of oxidative degradation (in 90% ACN with 3% H_2_O_2_) and standard curve. Table S1. ^1^H-NMR Spectral data for LCZ-D (600 MHz, CDCl_3_). Table S2. ^1^H-NMR Spectral data for LCZ-L (600 MHz, CDCl_3_).

## Abbreviations

The following abbreviations are used in this manuscript:

ARIA	Allergic Rhinitis and its Impact on Asthma
LCZ	Levocetirizine
LCZ-D	Levocetirizine decanoate
LCZ-L	Levocetirizine laurate
BB	Benzyl benzoate
BA	Benzyl alcohol
TW80	Polysorbate 80
DMSO	dimethylsulfoxide
PLE	Porcine liver esterase
BHT	Butylated hydroxytoluene
CO	Castor oil
MC	Chloromethane
SD	Sprague Dawley
TMS	Tetramethylsilane
MeOH	Methanol
DSC	Differential scanning calorimetry
Eq	Equivalent
HPLC	High-Performance Liquid Chromatography
EC_50_	Half Maximal Effective Concentration
HED	Human equivalent dose
