# Supplementary material for: Preparation and Evaluation of Long-Acting Injectable Levocetirizine Prodrug Formulation
Source: Pharmaceutics. 2025 Jun 21;17(7):806. doi: 10.3390/pharmaceutics17070806 (PMC12299168; doi:10.3390/pharmaceutics17070806)
Supplement: Supplementary file 1 [file pharmaceutics-17-00806-s001.zip › pharmaceutics-3697192-supplementary.pdf]

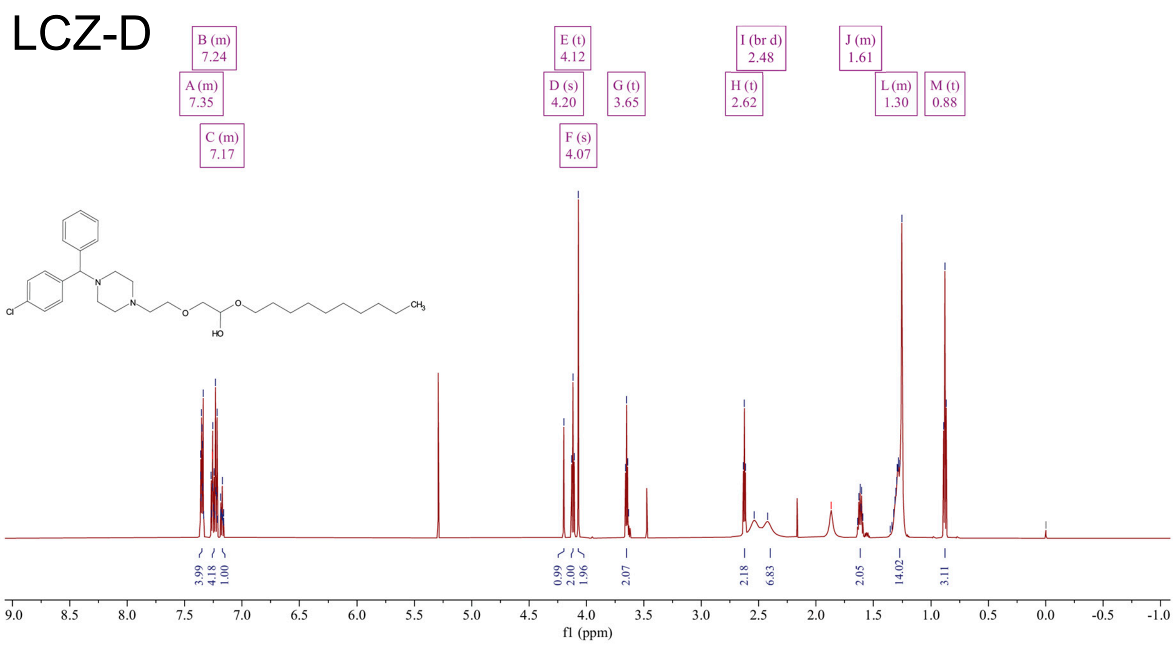

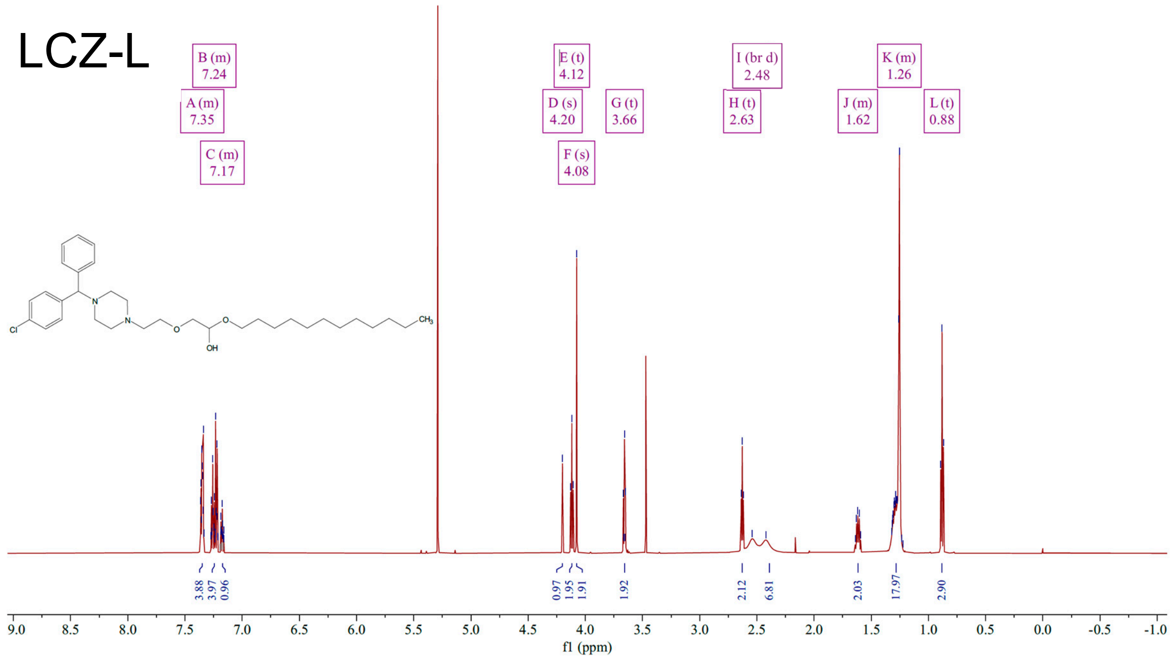

Figure S1. ^1H NMR spectra of LCZ-D and LCZ-L acquired in $CDCl_3$ at 600 MHz. Signal assignments for key protons are labeled.

Table S1. ^{1}H-NMR Spectral data for LCZ-D (600 MHz, $CDCl_3$).

δ (ppm)	Multiplicity	J (Hz)	Integration	Assignment
7.39 – 7.32	m	–	4H	Chlorophenyl
7.29 – 7.20	m	–	4H	Aromatic protons
7.20 – 7.14	m	–	1H	Aromatic proton
4.20	s	–	1H	C7 proton
4.12	t	6.8	2H	C1″ proton
4.07	s	–	2H	C17 proton
3.65	t	5.6	2H	C15 proton
2.62	t	5.6	2H	C14 proton
2.48	br d	Approx. 70	8H	Piperazine proton
1.65 – 1.58	m	–	2H	C2″ proton
1.37 – 1.20	m	–	14H	C3″–C9″
0.88	t	7.0	3H	C10″ proton

Table S2. ^{1}H-NMR Spectral data for LCZ-L (600 MHz, $CDCl_3$).

δ (ppm)	Multiplicity	J (Hz)	Integration	Assignment
7.38 – 7.32	m	–	4H	Chlorophenyl
7.29 – 7.20	m	–	4H	Aromatic protons
7.20 – 7.14	m	–	1H	Aromatic proton
4.20	s	–	1H	C7 proton
4.12	t	6.8	2H	C1″ proton
4.08	s	–	2H	C17 proton
3.66	t	5.7	2H	C15 proton
2.63	t	5.7	2H	C14 proton
2.48	br d	Approx. 70	8H	Piperazine proton
1.65 – 1.58	m	–	2H	C2″ proton
1.27 – 1.24	m	–	18H	C3″–C11″
0.88	t	7.0	3H	C12″ proton

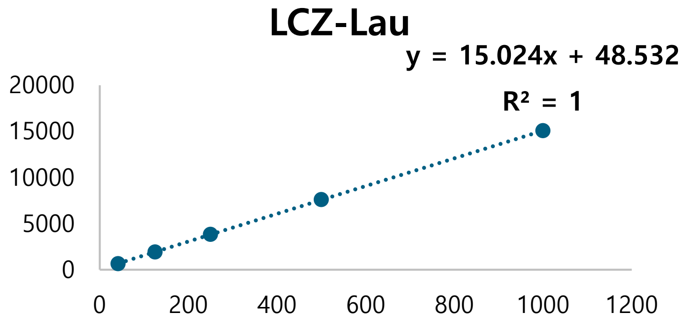

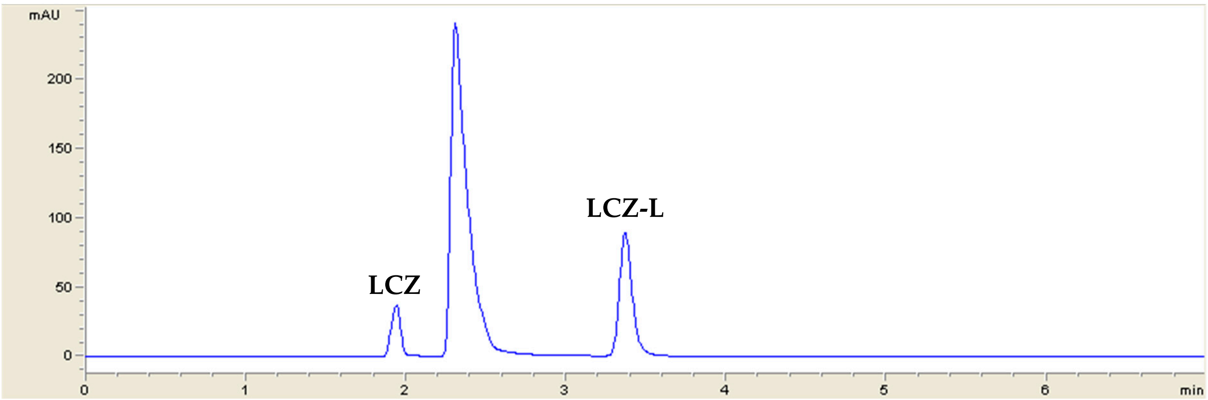

Figure S2. HPLC chromatogram of LCZ-L after 3 hours of oxidative degradation (in 90% ACN with 3% H_2O_2) and standard curve.
